# Endoscopic Full‐thickness Resection for Gastric Submucosal Tumor: A Technical Analysis Study (With Video)

**DOI:** 10.1002/deo2.70198

**Published:** 2025-09-08

**Authors:** Hitoshi Mori, Noriya Uedo, Satoki Shichijo, Muneshin Morita, Yushi Kawakami, Yasuhiro Tani, Hiroyoshi Iwagami, Muneaki Miyake, Taro Iwatsubo, Minoru Kato, Shunsuke Yoshii, Takashi Kanesaka, Koji Higashino, Tomoki Michida, Ryu Ishihara, Naoki Shinno, Hisashi Hara, Yoshitomo Yanagimoto, Kazuyoshi Yamamoto, Takeshi Omori, Hitoshi Yoshiji

**Affiliations:** ^1^ Department of Gastrointestinal Oncology Osaka International Cancer Institute Osaka Japan; ^2^ Department of Gastroenterology Nara Medical University Nara Japan; ^3^ Department of Gastroenterological Surgery Osaka International Cancer Institute Osaka Japan

**Keywords:** endoscopy, full‐thickness resection, gastrointestinal stromal tumors, stomach, submucosal tumor

## Abstract

**Background:**

Endoscopic full‐thickness resection (EFTR) is an effective treatment method for gastric submucosal tumors (SMTs). We aimed to perform a technical analysis of EFTR in gastric SMT and compare it with the outcome parameters.

**Method:**

Sixty‐one gastric SMTs from 60 patients were resected using EFTR. The indication criteria: size, 11–30 mm, connection to the muscularis propria on endoscopic ultrasonography, intraluminal growth type, no ulceration, and histologically evident or clinically suspicious gastrointestinal stromal tumors (GISTs). The following technical improvements were introduced during the study Periods 1–3: routine use of clip‐line traction (Periods 1–3); use of a plastic bag retriever (Periods 2–3); adaptation of the reopenable clip over‐the‐line method (ROLM, Period 3); implementation of no‐touch EFTR (Period 3); and elimination of submucosal injection (Period 3).

**Results:**

The endoscopic complete resection rate was 100%, with a similar tumor resection time (median, 50 min) throughout the periods. Specimen damage was less frequent after using the plastic bag retriever in Periods 2 and 3 (*p =* 0.001). In Period 3, ROLM required longer full‐thickness defect closure time (39 min, *p =* 0.011), but it provided secure closure and shortened the fasting days (*p =* 0.010). Histological diagnoses included 38 GISTs, 14 leiomyomas, and nine other pathologies. In Period 3, the implementation of no‐touch EFTR increased the resected specimen size (33 mm, *p* = 0.010) and improved the histological complete (R0) resection rate of the GISTs (13/13, 100%, *p =* 0.017).

**Conclusion:**

Several technical improvements significantly improved the outcomes of EFTR for gastric SMTs, warranting the external validation of this technique.

## 1 Introduction

Currently, the standard resection method for localized gastrointestinal stromal tumors (GISTs) is surgical resection [1, 2]. However, endoscopic resection of GISTs has rapidly evolved over the past decade [3]. To date, many studies on endoscopic full‐thickness resection (EFTR) for gastric submucosal tumors (SMTs) have been published [4, 5, 6, 7]; however, the detailed technique and associated outcomes of EFTR have not been well investigated. In this study, a technical analysis of EFTR in gastric SMT was performed.

## 2 Methods

### 2.1 Study Design and Setting

This retrospective study was conducted at a tertiary cancer center. The study period was divided into Period 1 (April 2018 to August 2020), Period 2 (September 2020 to June 2023), and Period 3 (July 2023 to April 2025), based on the timing of approval for advanced medical care and the implementation of the no‐touch EFTR concept.

### 2.2 Participants

The indication criterion for EFTR was initially patients with SMTs ≤3 cm without ulceration. From September 2020, the indication criteria further included: (1) size, 11–30 mm; (2) connection to the muscularis propria on endoscopic ultrasonography (EUS); (3) intraluminal growth type; (4) no ulceration; (5) histologically evident or clinically suspicious GISTs, such as those with irregular margins or enlargement; and (6) confirmation of indication by a multidisciplinary cancer board.

### 2.3 Pretreatment Diagnosis

Esophago‐gastro‐duodenoscopy and contrast‐enhanced computed tomography (CT) scans were performed for all patients. Pretreatment histological diagnosis was made using either a boring biopsy [8] or EUS‐fine needle aspiration biopsy.

### 2.4 Endoscopic Procedures

The EFTR procedures were performed under general anesthesia in the operating room. The patients were placed in the supine position. The main equipment used in the procedures is summarized in Table S1. Surgeons were on standby on call, but laparoscopic assistance was not provided in any of the cases.

**FIGURE 1:**
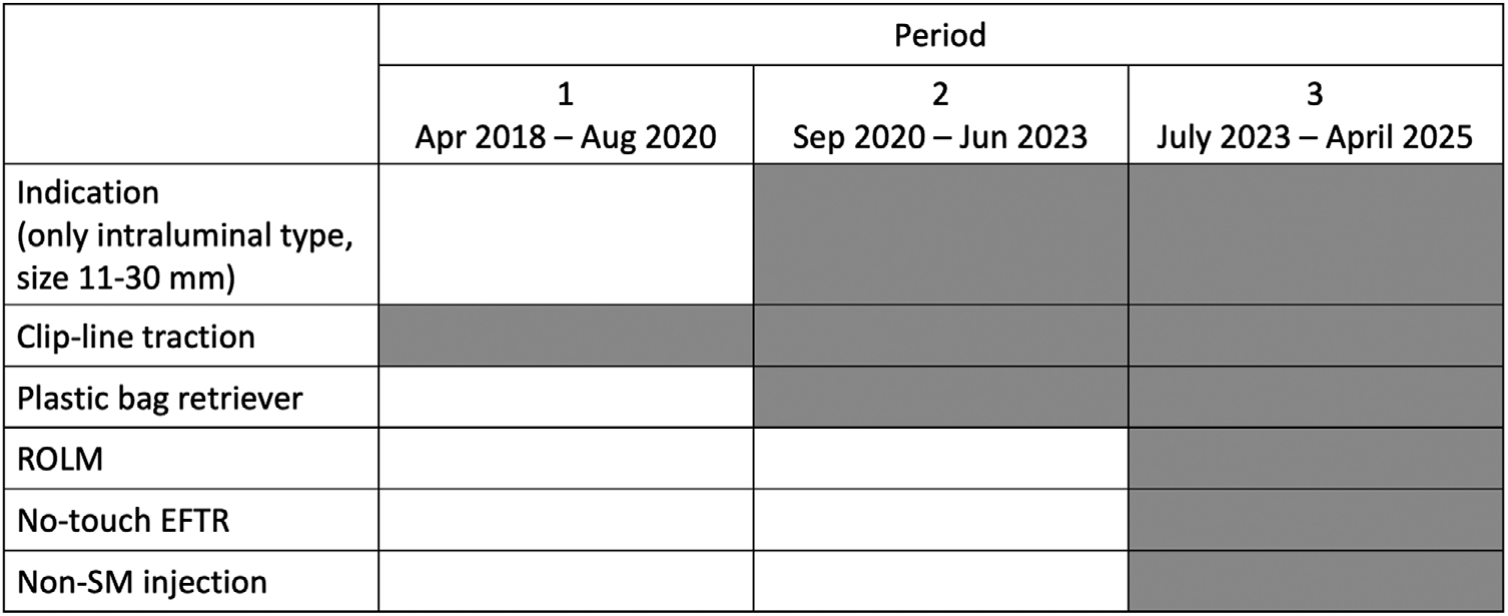
Technical refinements during the study period. ROLM: re‐openable clip over‐the‐line method, EFTR: endoscopic full‐thickness resection, and SM: submucosal.

**FIGURE 2:**
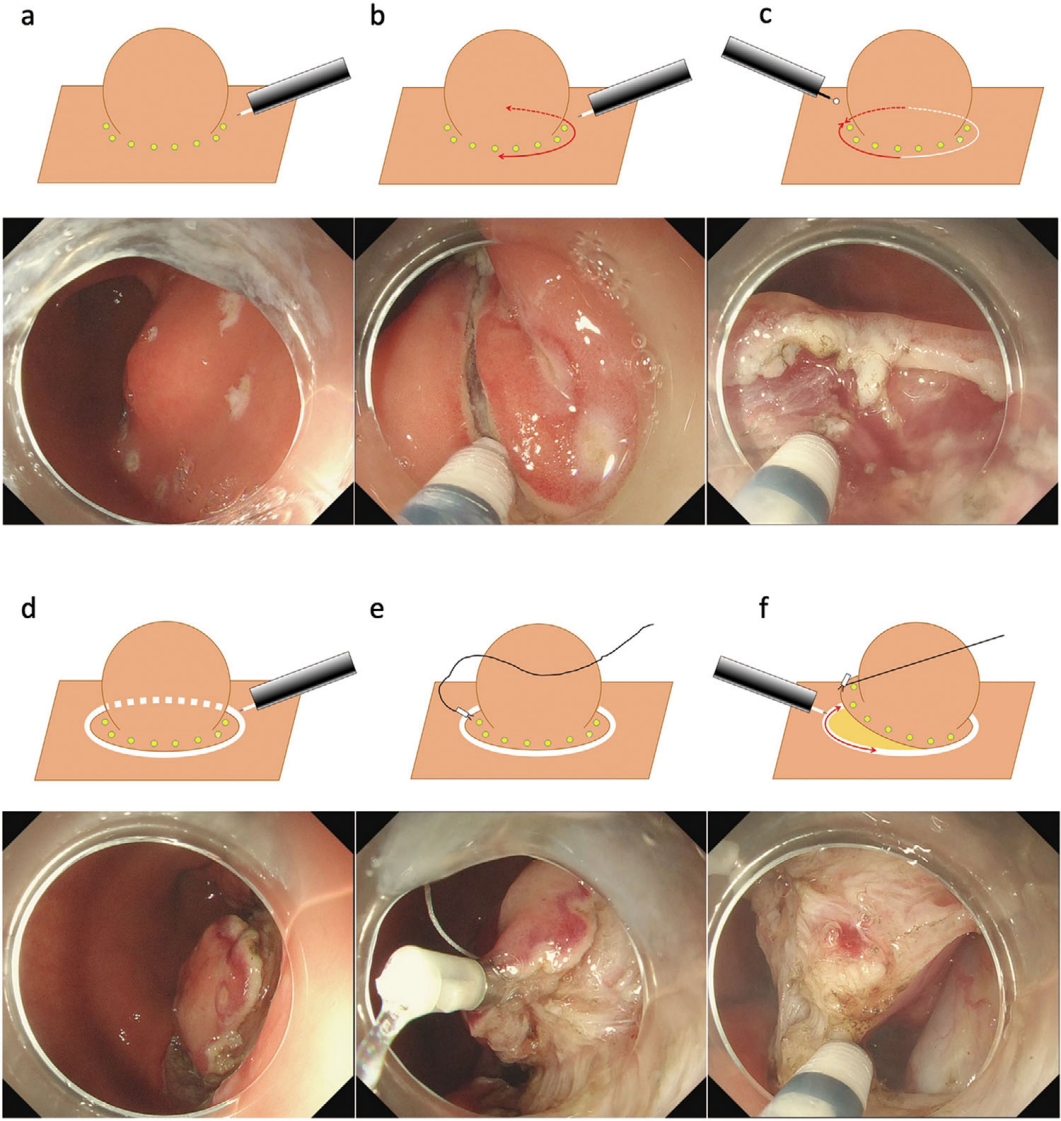
Schematic diagram of the latest endoscopic full‐thickness resection (EFTR) procedure. Marking dots were placed a few millimeters away from the base of the lesion (a). A mucosal incision was made outside the marking on the oral side (b). The mucosal incision was extended toward the anal side (c). The mucosal incision was deepened vertically toward the surface of the muscularis propria (d). Application of clip‐line traction (e). A muscular incision was made along the mucosal/submucosal incision line.

**FIGURE 3:**
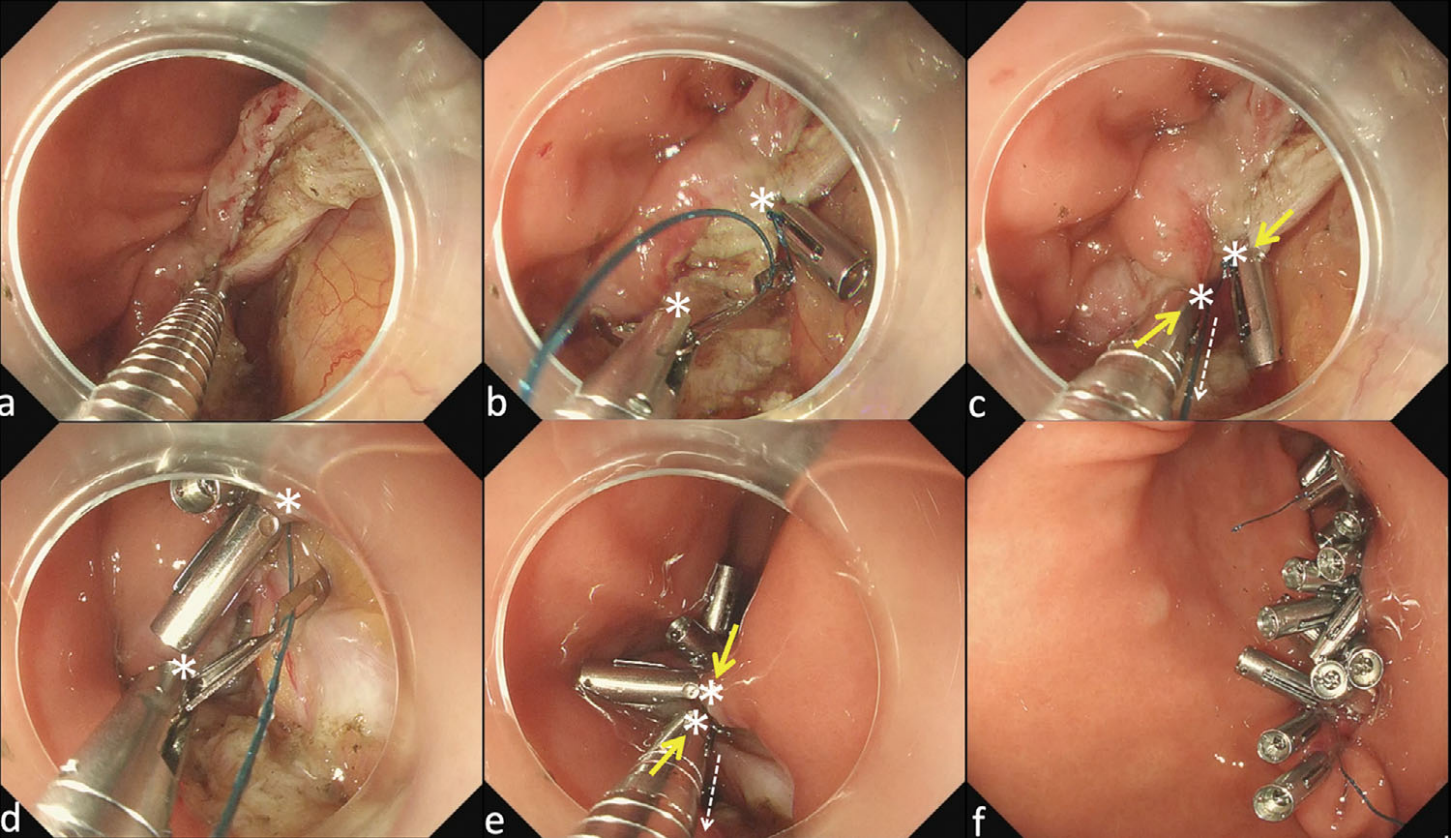
Reopenable clip over‐the‐line method. The first reopenable endo‐clip tied to the nylon line on the jaw was inserted through the working channel and deployed to the most distal edge of the full‐thickness defect (a). The second reopenable endo‐clip, threaded by a nylon line through a hole in the jaw, was roped through the working channel and was applied to the edge of the full‐thickness defect adjacent to the first endo‐clip (b). Before deployment of the second endo‐clip, the nylon line was pulled (white dotted line in c) to approximate (yellow arrows in c) the two clips (white asterisks in b and c). After a sufficient approximation of the edges of the full‐thickness defect, a second reopenable endo‐clip was deployed. Then, the reopenable endo‐clip, which was threaded by a nylon line, was applied to the contralateral edge of the full‐thickness defect (d). It was then approximated (yellow arrow in e) to the previous endo‐clip (white asterisks in d and e) by pulling the nylon line (white dotted line in e) before deployment. This procedure was repeated until the defects were completely closed. Finally, a line was cut 1 cm from the last reopenable endo‐clip using scissor forceps (FS‐3L‐1; Olympus, Tokyo, Japan).

Before performing the EFTR procedure, the main operator (Noriya Uedo) visited the leading hospital in Shanghai for a learning observership [9]. Thereafter, several technical refinements were introduced over time, as summarized in Figure 1. The latest procedural steps of EFTR for gastric SMT are as follows: (1) Circumferential marking with the needle‐type knife a few mm away from the base of the lesion, (2) Mucosal incision on the oral side outside the marking using a needle‐type knife (Flush Knife BT‐S, 2.0 mm), (3) Extension of the mucosal incision toward the anal side using a needle‐type or an insulated‐tip knife, (4) Deepening of the mucosal incision line to the surface of the muscularis propria (trimming) to form deep mucosal/submucosal groove, (5) Placement of a clip with line (3‐0 polyester suture line), (6) Muscular incision along the mucosal/submucosal groove, (7) Full‐thickness defect closure, and (8) Retrieval of the specimen (Figures 2 and 3, and Video S1).

Antibiotics were administered 30 min before and every 3 h during the EFTR procedure. When pneumoperitoneum occurred, abdominal paracentesis was performed with an intravenous catheter (Angiocath IV catheter, 14G) connected to a 20‐mL syringe filled with normal saline, inserted on the left side opposite the McBurney point.

Routine second‐look endoscopy was not performed.

### 2.5 Technical Refinements

#### 2.5.1 Clip‐line Traction

Clip‐line traction was initially used only occasionally during EFTR procedures. However, it was found to be useful for identifying incision lines during muscle incision and the dissection plane during sub‐tumoral tissue dissection. Consequently, it was routinely used during Period 1.

#### 2.5.2 Plastic Bag Retriever

When a snare or retrieval net was used to collect the resected specimens, the specimens were often damaged when passing through the esophagogastric junction. Therefore, starting in December 2021, a drawstring‐type plastic bag retriever was used to extract the resected specimens.

#### 2.5.3 Reopenable Clip Over‐the‐Line Method

Initially, full‐thickness defects were closed using the purse‐string closure method with endo‐loops and endo‐clips [10]. In this method, an endo‐loop was attached to the mucosal edge of a full‐thickness defect using multiple endo‐clips, and the wound was closed by tightening the endo‐loop. However, starting in February 2023, the reopenable clip‐over‐the‐line method (ROLM) using a 3‐0 nylon suture line and endo‐clips was adopted (Figure 3) [11, 12]. This method provides a strong grasping and closure force because the endo‐clips bite all the layers of the gastric wall.

#### 2.5.4 No‐Touch EFTR

Because ROLM enables the closure of a large full‐thickness wound securely, we implemented the no‐touch EFTR concept in July 2023 [13]. In this method, the mucosa outside the tumor was incised circumferentially, and full‐thickness layers were cut vertically to the muscularis propria along the mucosal incision line without touching the tumor.

#### 2.5.5 Injection Solution

Initially, a sodium hyaluronate solution was injected into the submucosa for mucosal incision and submucosal dissection, as in endoscopic submucosal dissection (ESD). However, a submucosal injection was not performed from May 2023.

### 2.6 Post‐EFTR Management

Proton pump inhibitors were administered from the day of treatment until postoperative day (POD). A nasogastric tube was placed postoperatively. Blood tests and physical examinations were performed on POD 1. If the patient had no or mild abdominal pain and a fever <38°C on POD 1, drinking water and a liquid diet were started from POD 2. If the patient presented with moderate or greater abdominal pain or a fever ≥38°C, fasting was continued. After improvement in abdominal pain, the patient started drinking water and resumed a liquid diet the following day.

### 2.7 Definition of Variables

The tumor location was defined according to the Japanese Classification of Gastric Carcinomas [14]. The intraluminal type was defined as the tumor epicenter above the midline of the muscularis propria on EUS and CT images. The time for lesion resection was measured from the marking to the detachment of the lesion. Endoscopic complete resection was defined as ER0: tumor resected en bloc with no endoscopic evidence of residual tumor; otherwise, endoscopic findings of residual tumor or piecemeal resection of the tumor were defined as ER1. Muscular preservation was defined as preservation of the muscularis propria at the bottom of the wound. The time to defect closure was measured from the detachment of the lesion to the completion of defect closure. Specimen damage was defined as a macroscopic fragmentation during retrieval. Adverse events were graded according to the Clavien–Dindo classification [15].

### 2.8 Histological Diagnosis

**FIGURE 4:**
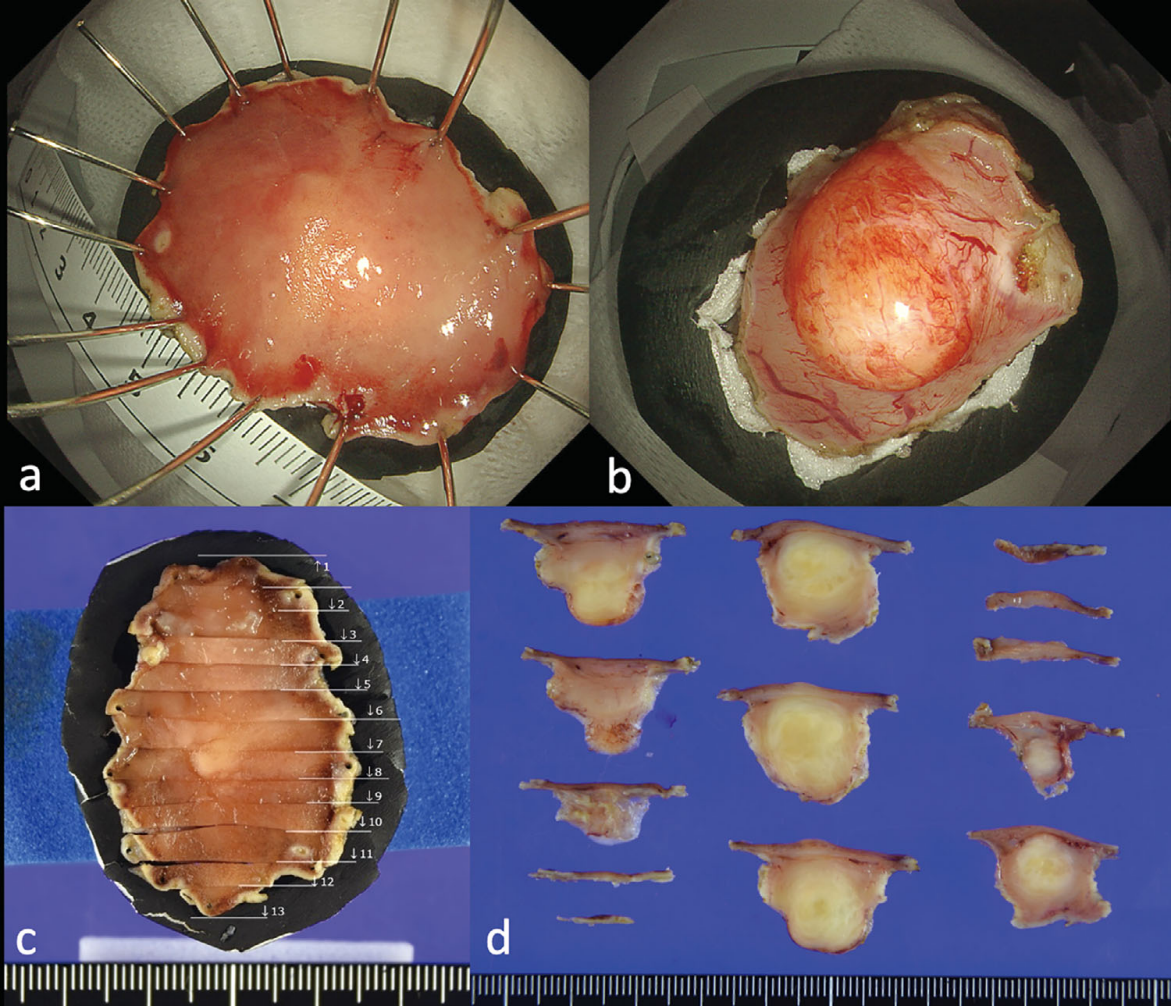
Retrieved specimen. The retrieved specimen was pinned onto a Styrofoam plate (a: mucosal side, b: serosal side). After fixation, the specimens were sectioned at 3 mm intervals (c and d).

After fixation with 10% formalin, the specimen was cut into 3 mm sections to examine the tumor's maximum diameter and resection margins (Figure 4). Histopathological findings from hematoxylin and eosin staining and immunohistochemical staining were used to diagnose GIST. For GIST, the tumor risk was classified based on the modified Fletcher classification [16]. A clear histological margin of the entire tumor in all sections was defined as histological complete resection (R0); otherwise, an unclear or positive histological margin was defined as non‐R0.

### 2.9 Statistical Analysis

Data are expressed as medians (interquartile ranges) for continuous variables and real numbers (proportions) for categorical variables. Group differences were compared using the chi‐square test or Fisher's exact probability test for categorical variables and the Kruskal‐Wallis test for continuous variables. The level of statistical significance was set at *p* < 0.05.

## 3 Results

### 3.1 Background Data

**FIGURE 5:**
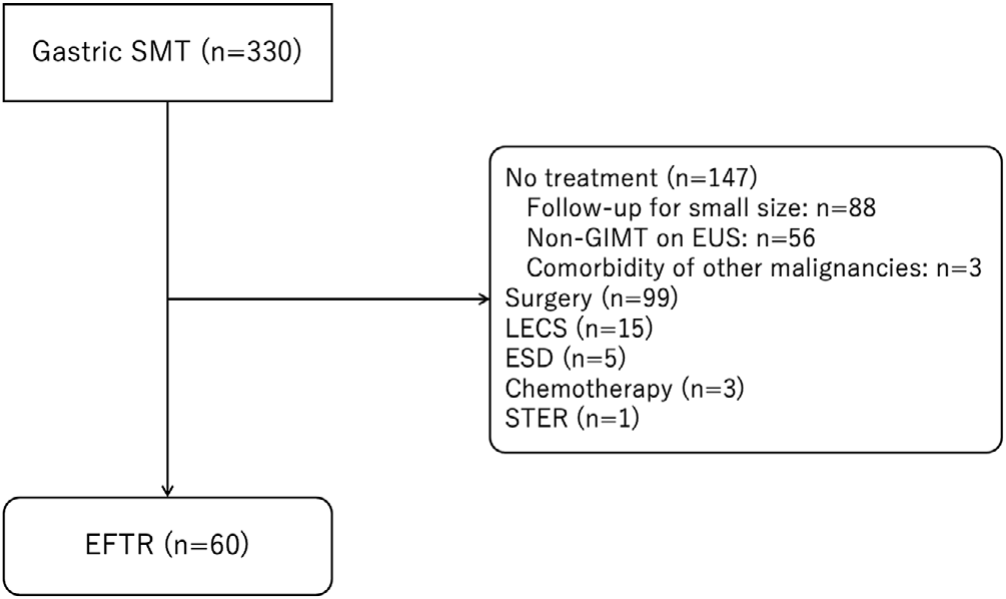
Participants flow. ESD: endoscopic submucosal dissection, EUS: endoscopic ultrasonography, GIMT: gastrointestinal mesenchymal tumor, GIST: gastrointestinal stromal tumor, LECS: laparoscopic endoscopic cooperative surgery, POET: peroral endoscopic tumor resection, and SMT: submucosal tumor.

During the study period, 330 patients with gastric SMT visited our institute. Among them, 60 patients with 61 lesions were treated with EFTR and analyzed (Figure 5). In Period 1, only patients seen by the main investigator (N.U.) were enrolled, but in Periods 2 and 3, patients referred to our hospital, assessed for eligibility, and who fulfilled the inclusion criteria were consecutively enrolled.

A total of 16, 22, and 23 lesions were treated during Periods 1, 2, and 3, respectively (Table 1). From Period 2 onwards, the indication for EFTR was restricted to intraluminal growth (*p* = 0.012).

**TABLE 1:** Background data (clinical).

	Total (*n* = 61)	Period			
		1 (*n* = 16)	2 (*n* = 22)	3 (*n* = 23)	*p*‐Value
Age^‡^	60 (48–71)	69 (52–71)	53 (43–62)	65 (49–77)	0.031
Sex^‡^					0.393
Men	30 (50)	9 (60)	12 (55)	9 (39)	
Women	30 (50)	6 (40)	10 (45)	14 (61)	
Pretreatment lesion size (mm)	18 (15–25)	22 (13–29)	18 (16–21)	15 (13–25)	0.577
Longitudinal lesion location					0.070
Upper third	38 (62)	10 (63)	9 (41)	19 (83)	
Middle third	15 (25)	4 (25)	9 (41)	2 (8.7)	
Lower third	8 (13)	2 (13)	4 (18)	2 (8.7)	
Circumferential lesion location					0.104
Anterior wall	15 (25)	2 (13)	7 (32)	6 (26)	
Lesser curve	9 (15)	1 (6.3)	6 (27)	2 (8.7)	
Post wall	24 (39)	7 (44)	5 (23)	12 (52)	
Greater curve	13 (21)	6 (38)	4 (18)	3 (13)	
Growth type					0.012
Intraluminal	58 (95)	13 (81)	22 (100)	23 (100)	
Extraluminal	3 (4.9)	3 (19)	0	0	
Method of biopsy					0.617
Boring biopsy	35 (57)	8 (50)	11 (50)	16 (70)	
EUS‐FNA	11 (18)	4 (25)	4 (18)	3 (13)	
Not done	15 (25)	4 (25)	7 (32)	4 (17)	
Pretreatment histology					0.082
GIST	20 (33)	7 (44)	9 (41)	4 (17)	
Leiomyoma	3 (4.9)	0	2 (9.0)	1 (4.4)	
Schwannoma	1 (1.6)	1 (6.3)	0	0	
Glomus tumor	1 (1.6)	1 (6.3)	0	0	
Non‐neoplastic tissue	20 (33)	3 (19)	4 (18)	13 (57)	
Not done	16 (26)	4 (25)	7(32)	5 (22)	
Indication					0.299
Histological GIST	20 (33)	7 (44)	9 (41)	4 (17)	
Enlargement	29 (48)	5 (31)	9 (41)	14 (61)	
Irregular margin	12 (20)	4 (25)	4 (18)	5 (22)	

^‡^ Data for 60 patients.

The procedural background data are summarized in Table 2. In Periods 1 and 2, submucosal injection was used for most lesions (37/38, 97%); however, it was used in four (17%) lesions in Period 3 (*p*<0.001). Clip‐line traction was used for 52 (85%) lesions during all periods. Complete muscle resection was performed for 54 (89%) lesions; however, only 18 (30%) patients required abdominal paracentesis. In Periods 1 and 2, the full‐thickness defect was closed using the purse string closure method for 14 (88%) and 17 (77%) lesions, respectively. However, it was closed using ROLM for 22 (96%) lesions in Period 3 (*p*<0.001). In Period 1, 13 lesions (81%) were retrieved using retrieval nets, but plastic bag retrievers were used for 16 (83%) and 19 (83%) lesions in Periods 2 and 3, respectively.

**TABLE 2:** Background data (procedural).

	Total	Period			
		1 (*n* = 16)	2 (*n* = 22)	3 (*n* = 23)	*p*‐Value
ESD devices					0.268
Flush knife + IT knife	57 (93)	14 (88)	22 (100)	21 (91)	
Flush knife	4 (6.6)	2 (13)	0	2 (8.7)	
Injection solution					<0.001
Sodium hyaluronate	26 (43)	15 (94)	11 (50)	0	
Normal saline	15 (25)	1 (6.3)	10 (45)	4 (17)	
None	20 (33)	0	1 (4.5)	19 (83)	
Clip‐line traction					0.851
Used	52 (85)	14 (88)	18 (82)	20 (87)	
Not used	9 (15)	2 (12)	4 (18)	3 (13)	
Muscle preservation					0.129
Yes	7 (11)	4 (25)	1 (4.5)	2 (8.7)	
No	54 (89)	12 (75)	21 (95)	21 (91)	
Abdominal paracentesis^‡^					0.944
Done	18 (30)	4 (27)	7 (32)	7 (30)	
Not done	42 (70)	11 (73)	15 (68)	16 (70)	
Defect closure method					<0.001
Purse‐string closure	31 (51)	14 (88)	17 (77)	0	
ROLM	26 (43)	0	4 (18)	22 (96)	
OTSC	2 (3.3)	1 (6.3)	1 (4.5)	0	
Not done	2 (3.3)	1 (6.3)	0	1 (4.4)	
Retrieval device					<0.001
Snare	2 (3.3)	1 (6.3)	0	1 (4.4)	
Retrieval net	21 (34)	13 (81)	6 (27)	2 (8.7)	
Plastic bag retriever	35 (57)	0	16 (83)	19 (83)	
Grasping forceps	3 (4.9)	2 (13)	0	1 (4.4)	

OTSC, over‐the‐scope clip; ROLM, reopenable‐clip over the line method.

^‡^ Data for 60 patients.

### 3.2 Outcome Data

Clinical outcome data are listed in Table 3.

**TABLE 3:** Outcome data (clinical).

	Total	Period			
		1 (*n* = 16)	2 (*n* = 22)	3 (*n* = 23)	*p*‐Value
Time for lesion resection (min)	50 (38–77)	57 (30–77)	52 (40–85)	49 (36–77)	0.871
Resected specimen size (mm)	28 (21–36)	25 (15–35)	25 (20–31)	33 (27–40)	0.010
En bloc resection	61 (100)	16 (100)	22 (100)	23 (100)	NA
Endoscopic complete resection					NA
ER0	61 (100)	16 (100)	22 (100)	23 (100)	
ER1	0	0	0	0	
Time for defect closure	31 (19–41)	16 (12–34)	29 (24–34)	38 (27–46)	0.018
Specimen damage during retrieval					0.010
Present	7 (11)	5 (31)	0	2 (8.7)	
Absent	54 (89)	11 (69)	22 (100)	21 (91)	
Adverse events (Clavien‐Dindo ≥Grade III)*	1 (1.7)	0	1 (4.5)	0	0.416
Days to resume meal (POD)^‡^					0.000
2	20 (33)	4 (27)	2 (9.1)	14 (61)	
3	25 (42)	3 (20)	14 (64)	8 (35)	
≥4	15 (25)	8 (53)	6 (27)	1 (4.4)	
Days to discharge (POD)^‡^					0.017
≤5	19 (32)	3 (20)	3 (14)	13 (57)	
6	23 (38)	7 (50)	12 (55)	4 (17)	
≥7	18 (30)	5 (33)	7 (32)	6 (26)	

ER0, endoscopic complete resection; ER1, endoscopic incomplete resection; POD, post‐operation day

^‡^ Data for 60 patients.

#### 3.2.1 Tumor Resection

The time for lesion resection was 50 (38–77) min, which did not differ between the treatment periods. The resected specimen size was larger after the implementation of no‐touch EFTR [Period 3: 33 (27–40) mm] than before [Period 1: 25 (15–35) mm; Period 2: 25 (20–31) mm, *p* = 0.010]. Endoscopic complete resection was achieved for all lesions (100%) throughout the period.

#### 3.2.2 Defect Closure

The time required for full‐thickness defect closure was longer in Period 3 [38 (27–46) min] with ROLM than in Periods 1 [16 (12–34) min] and 2 [29 (24–34) min] (*p* = 0.018). There was no case of incomplete closure requiring surgical conversion.

#### 3.2.3 Specimen Damage During Retrieval

The specimen damage rate was lower in Periods 2 (*n* = 0) and 3 (*n* = 2, 10%) with the plastic bag retriever than in Period 1 (n = 5, 31%, *p* = 0.010), when snares or retrieval nets were used. In Period 3, two lesions resulted in specimen damage. They were collected using a retrieval net because the plastic bag retriever was not available at the time due to a production recall.

#### 3.2.4 Adverse Events

No intraoperative complications were observed during the study period. One patient in Period 2 developed delayed perforation on POD 1, which was successfully managed with endoscopic treatment.

#### 3.2.5 Post‐procedural Course

Only four (27%) and two (9.1%) patients started meals from POD 2 in Periods 1 and 2, respectively. In contrast, after the implementation of ROLM, more than half of the patients (*n* = 14, 61%) were able to resume meals from POD 2 in Period 3 (*p* = 0.001). Hospitalization period was similarly shorter in Period 3 than in Periods 1 and 2 (*p* = 0.017).

#### 3.2.6 Histology

Histological diagnoses included 38 GIST, 14 leiomyomas, five schwannomas, one glomus tumor, and four other pathologies (Table 4). The R0 rate for GIST was significantly improved after the implementation of no‐touch EFTR (Period 3: 100%) compared to before (Period 1: 58%; Period 2: 54%; *p* = 0.017).

**TABLE 4:** Outcome data (histological).

	Total	Period			
		1 (*n* = 16)	2 (*n* = 22)	3 (*n* = 23)	*p*‐Value
Histological lesion size (mm)		23 (16–26)	20 (17–25)	23 (15–34)	0.679
Histology					0.179
GIST	38 (62)	12 (75)	13 (59)	13 (57)	
Leiomyoma	14 (23)	1 (6.3)	4 (18)	9 (39)	
Schwannoma	5 (8.2)	1 (6.3)	3 (14)	1 (4.3)	
Glomus tumor	1 (1.6)	1 (6.3)	0	0	
Others	3 (4.9)	1 (6.3)	2 (9)	0	
Risk category in GIST^§^					0.361
Very low	16 (42)	3 (25)	5 (38)	8 (62)	
Low	14 (37)	4 (33)	5 (38)	5 (38)	
Intermediate	4 (11)	3 (25)	1 (7.7)	0	
High	4 (11)	2 (17)	2 (15)	0	
Histological complete resection in GIST^§^					0.017
R0	27 (71)	7 (58)	7 (54)	13 (100)	
Non‐R0	11 (29)	5 (42)	6 (46)	0	

GIST, gastrointestinal stromal tumor; R0, histological complete resection; R1, histological incomplete resection; RX; histological resection margin unclear.

^§^ Data for 36 lesions.

## 4 Discussion

This study demonstrated improved outcomes with EFTR for gastric SMT following several technical refinements.

**FIGURE 6:**
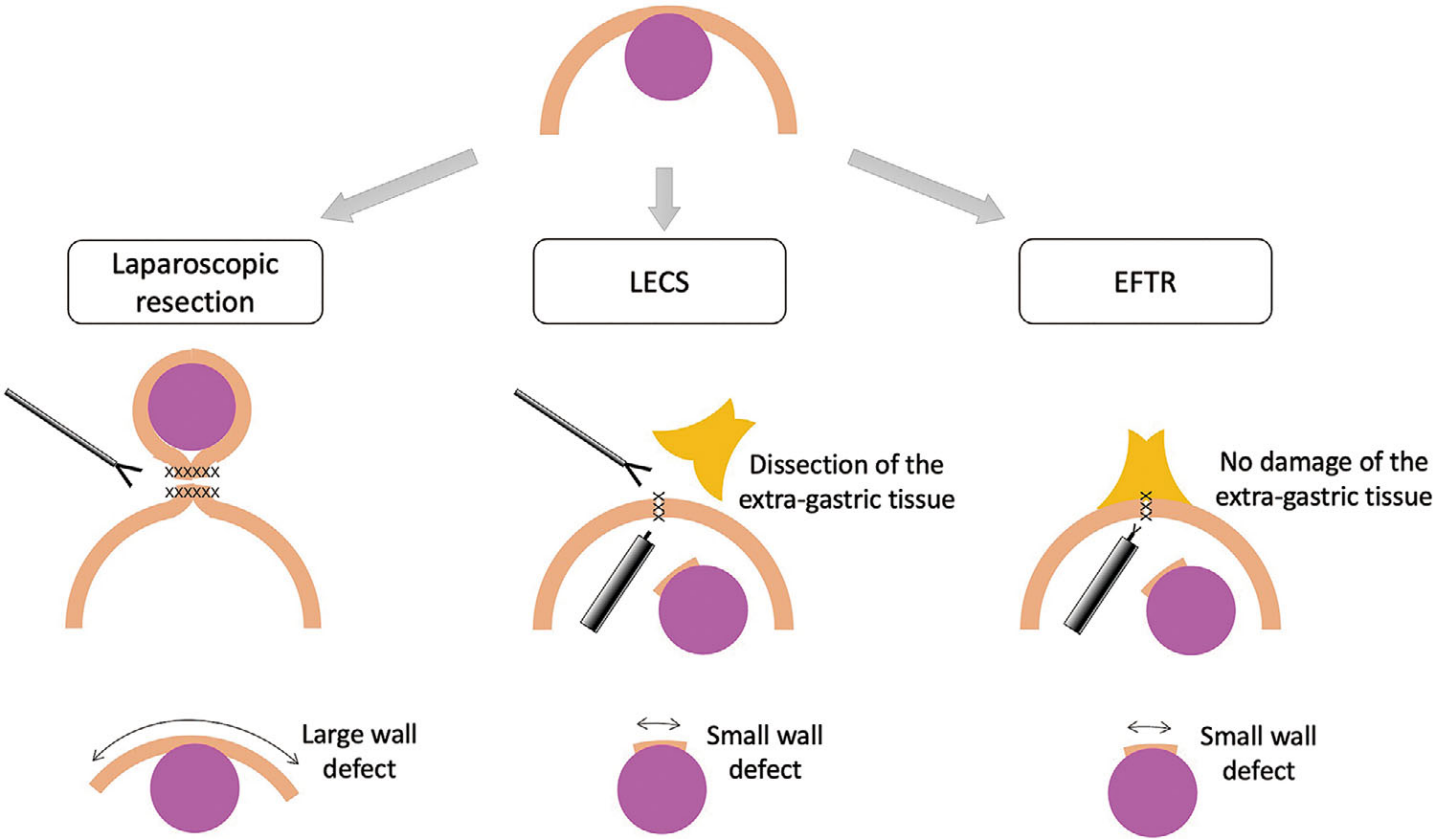
Clinical benefits of endoscopic full‐thickness resection (EFTR).

In Period 1, the indication for EFTR was determined by consensus among endoscopists [17]. However, during the approval process for EFTR as an advanced medical care by the Ministry of Health, Labour and Welfare in Japan, it was necessary to clarify its benefits despite the availability of the established surgical treatment. Extraluminal GISTs are easier to remove surgically, but intraluminal GISTs can be difficult to identify. Moreover, laparoscopic excision may require extensive resection margins (Figure 6), and gastrectomy may be required if the lesion is located near the cardia or pylorus, although GISTs are oncologically amenable to local resection since they lack infiltrative growth or regional lymph node metastasis. To address this issue, Laparoscopic and endoscopic cooperative surgery (LECS) has been developed [18]. However, in LECS, when extra‐gastric tissue is attached outside the gastric wall, it must be dissected to close the defect. Thus, EFTR can minimize gastric wall resection and avoid extra‐gastric tissue dissection, offering a minimally invasive treatment. The indication criteria for lesion size, initially set at ≤3 cm, was changed to a maximum of 11–30 mm to avoid overtreatment of small GISTs. The revision of the indication criteria had little effect on the clinical outcomes between Periods 1 and 2. However, it was important to gain consensus among surgeons to perform EFTR for gastric SMTs in our institute.

The American Society for Gastrointestinal Endoscopy guidelines divide the EFTR technique into exposed and non‐exposed methods [19]; the former is subdivided into tunneled [20, 21] and non‐tunneled techniques. In our experience, the non‐tunneled exposed technique was preferred for EFTR of gastric GISTs because of difficulties in creating a tunnel in certain locations (e.g., the fornix) and the increased risk of tumor damage with the tunneled technique. Chiu et al. also found that non‐tunneled EFTR achieved a higher en bloc resection rate for GISTs [22]. We consider that exposed non‐tunneled EFTR is more suitable for endoscopic resection of GISTs than tunneled EFTR, offering high en bloc and R0 resection rates, irrespective of tumor location.

In Periods 1 and 2, a mucosal incision was made on the edge of the tumor; the submucosa was dissected, and the muscularis propria was incised to minimize full‐thickness defects [10]. This was because, in the purse‐string closure method, the size of the full‐thickness defects closed at one time was limited by the size of the endo‐loop, and multiple closures were necessary to close the large defects. However, tissue dissection near the tumor made the diagnosis of R0 difficult and sometimes caused capsule injury. After the implementation of no‐touch EFTR with ROLM (Period 3), the lesions were resected with marginal tissues because a large full‐thickness defect could be closed securely. Thus, an excellent R0 rate of 100% was obtained.

Although the size of the resected specimen increased in Period 3, the time required for lesion resection did not increase significantly. It is possible that the learning curve contributed to the reduction in resection time; however, we assumed that the absence of submucosal injections might have shortened the excision time. Without submucosal injection, the submucosa remained thin, and the time to reach the surface of the muscular layer was shorter than that with submucosal injection. Other benefits of not using submucosal injection included easier recognition of the tumor boundary during mucosal incision and easier grasping of the edge of the full‐thickness defect by clips during ROLM. Mucosal incision without submucosal injection sometimes caused unintended muscle incision, but such small perforations did not affect the subsequent steps of the EFTR procedure. The only disadvantage of omitting submucosal injection was a slight increase in the incidence of bleeding during mucosal incision, but we considered that this was outweighed by the advantages mentioned above.

In Periods 1 and 2, many patients who underwent the purse‐string closure method for full‐thickness defects experienced epigastric pain for several days, and resumed meals on POD 3 or later. However, after the implementation of ROLM in Period 3, most patients experienced only mild and limited epigastric pain for a few days [12], enabling the majority to resume meals from POD 2. We assume that a gap may have formed between the endoclips in the purse‐string closure method, as it only approximates the mucosa, resulting in minor leakage of gastric contents and localized peritonitis. In contrast, ROLM has the potential to achieve watertight closure, including the muscle layer, reduce inflammation around the full‐thickness wound, and shorten the fasting and hospitalization periods.

No‐touch EFTR with ROLM significantly improved the R0 rate for GISTs in Period 3 (*p* = 0.022). The management of non‐R0 with macroscopically complete resection is controversial. Regarding surgical treatment, some reports suggest the significance of R0 status as a prognostic indicator [23, 24]. Still, others indicate no significant association between the R0 status and recurrence‐free or overall survival in patients undergoing macroscopically complete surgical excision [25, 26, 27]. Zhu et al. suggested that no statistical increase in recurrence after endoscopic resection of GISTs in patients who underwent R1 but ER0 resection. However, there was one recurrence in the R1 cases (1/286) and none in the R0 cases (0/85) [28]. In surgical resection of GISTs, R0 excision is the goal in any case [1]. Our technical improvements complied with this policy.

This study had some limitations. First, because this was a retrospective, single‐center study, the results require external validation through prospective studies at other institutions. Second, the study evaluated only short‐term outcomes, as the primary aim was to analyze the endoscopic techniques of EFTR. Longer follow‐up studies are needed to assess recurrence and survival in patients with high‐risk GISTs or those with non‐R0 resections. Third, all procedures at our institution were performed by experienced endoscopists; therefore, the outcomes may have been influenced by operator expertise. Based on our experience, we believe that endoscopists with sufficient experience in gastric ESD and emergency hemostasis can replicate our results.

In conclusion, several technical improvements significantly reduced specimen damage during retrieval, shortened the fasting and hospitalization period, and improved the R0 rate of EFTR for GISTs, warranting external validation of this technique.

## Conflicts of Interest

Noriya Uedo received honoraria for lectures from Olympus Co. Ltd., FUJIFILM Co. Ltd., Boston Scientific Japan, Daiichi‐Sankyo Co. Ltd., Takeda Pharmaceutical Co. Ltd., EA Pharma Co. Ltd., Otsuka Pharmaceutical Co. Ltd., AstraZeneca Co. Ltd., Miyarisan Pharmaceutical Co. Ltd., and AI medical service Co Ltd.; Satoki Shichijo received honoraria for lectures from FUJIFILM Co Ltd., Boston Scientific Japan, EA Pharma Co Ltd, AstraZeneca Co. Ltd., AstraZeneca, Co. Ltd., Daiichi‐Sankyo Co Ltd., AI medical service Co Ltd., Zeria Pharmaceutical Co., Ltd., and Janssen Pharmaceutical Co., Ltd.; Ryu Ishihara received honoraria from Olympus Co. Ltd., FUJIFILM Co Ltd., Daiichi‐Sankyo Co Ltd., Miyarisan Pharmaceutical Co. Ltd., AI medical service Co Ltd., AstraZeneca Co. Ltd., MSD Co Ltd., and Ono Pharmaceutical Co Ltd. and other authors have no conflict of interest to disclosed.

## Ethics Statement

The study protocol was approved by the Institutional Review Board (No. 25008). Before EFTR, all patients provided written informed consent for the procedure and comprehensive consent for the use of medical data in future research. Specific consent for study participation was informed on the website and obtained by an opt‐out method. Study Registration was not required, as this was a retrospective observational study. This study enrolled human subjects and was not an animal study.

## Supporting information




**VIDEO S1**: No‐touch endoscopic full‐thickness resection using the reopenable clip over‐the‐line method for defect closure.

**TABLE S1**: Equipment for EFTR.
