# Supplementary material for: Endoscopic Full‐thickness Resection for Gastric Submucosal Tumor: A Technical Analysis Study (With Video)
Source: DEN Open. 2025 Sep 8;6(1):e70198. doi: 10.1002/deo2.70198 (PMC12417311; doi:10.1002/deo2.70198)
Supplement: Supplementary file 2 — TABLE S1: Equipment for EFTR. [file DEO2-6-e70198-s002.docx]

Supplementary Table 1. Equipment for EFTR

|  | Generic name | Product name, number | Manufacturer |
| --- | --- | --- | --- |
| Endoscopy system | Light source | CLV-290 | Olympus Medical systems, Co. Ltd., Tokyo |
|  | Processor | CV-290 | Olympus Medical systems, Co. Ltd., Tokyo |
|  | Endoscopes | GIF-Q260J  GIF-H290T  GIF-2TQ260M | Olympus Medical systems, Co. Ltd., Tokyo |
| Hood | Distal attachment | D-201-13404  D-201-11804 | Olympus Medical systems, Co. Ltd., Tokyo |
| ESD knives | Insulated tip knife | IT knife 2, KD-611L | Olympus Medical systems, Co. Ltd., Tokyo |
|  | Needle type knife | Flush knife BT-S, 2.0 mm, DK-2620-B20S | Fujifilm, Co. Ltd., Tokyo |
| Hemostatic forceps | Hot biopsy forceps | Radial Jaw 4, Hot Biopsy Forceps | Boston Scientific, Marlborough, MA, USA |
|  | Hemostatic forceps | Coagrasper G, FD-412LR | Olympus Medical systems, Co. Ltd., Tokyo |
| Electrosurgical unit | High-frequency electric generator | VIO300D | Erbe Elektromedizin GmbH, Tubingen |
|  |  | VIO 3 | Erbe Elektromedizin GmbH, Tubingen |
| Injection needle | Endoscopic puncture needles | 25G, 4 mm, IFH, 38825000 | TOP Co., Ltd., Tokyo |
| Injection solution | Hyaluronic acid | MucoUp, 0.4% sodium hyaluronate | Boston Scientific Corporation, Marlborough, MA |
| Retriever | Plastic bag retriever | ENDO CARRY, Large type, 29112010 | Hakko Co., Ltd., Nagano |
| Retrieval net | Loop net | Netis, MED-132-NET | Meditalia S.R.L., Palermo |
| Suture line | Line for traction | 3-0 polyester surgical thread | Natsume Seisakusho Co., Ltd., Tokyo |
|  | Line for ROLM | 3-0 nylon surgical thread | Akiyama Medical Co., Ltd., Tokyo |
| Endoclips | Endoclip for traction | EZ clip, HX-610-090 | Olympus Medical systems, Co. Ltd., Tokyo |
|  | Endoclip for ROLM | Sureclip,  ROCC-F-26-195-C | Micro-tech, Co., Ltd., Nanjing |
| Endo-loop | Loop ligation device | HX-400U-30 | Olympus Medical systems, Co. Ltd., Tokyo |
| Paracentesis needle | Intravenous catheter | Angiocath IV  Catheter, 14G | Becton Dickinson, Franklin Lakes, NJ, USA |
